# Whole genome molecular phylogeny of large dsDNA viruses using composition vector method

**DOI:** 10.1186/1471-2148-7-41

**Published:** 2007-03-15

**Authors:** Lei Gao, Ji Qi

**Affiliations:** 1 The Institute of Theoretical Physics, Academia Sinica, Beijing 100080, China; 2 The T-Life Research Center, c/o Department of Physics, Fudan University, Shanghai 200433, China; 3 Center for Comparative Genomics and Bioinformatics, Penn State University, University Park, PA 16802, USA

## Abstract

**Background:**

One important mechanism by which large DNA viruses increase their genome size is the addition of modules acquired from other viruses, host genomes or gene duplications. Phylogenetic analysis of large DNA viruses, especially using methods based on alignment, is often difficult due to the presence of horizontal gene transfer events. The recent composition vector approach, not sensitive to such events, is applied here to reconstruct the phylogeny of 124 large DNA viruses.

**Results:**

The results are mostly consistent with the biologist's systematics with only a few outliers and can also provide some information for those unclassified viruses and cladistic relationships of several families.

**Conclusion:**

With composition vector approach we obtained the phylogenetic tree of large DNA viruses, which not only give results comparable to biologist's systematics but also provide a new way for recovering the phylogeny of viruses.

## Background

Viruses are small, infectious, obligate intracellular parasites that are capable of replicating themselves within their host cells. They are even smaller than the smallest elementary biosystem, yet still possess some properties of living systems such as having a genome and the ability to adapt to changing environments. However, viruses cannot capture and store free energy and they are not functionally active outside their host cell [1].

Traditionally, viruses were characterized by morphological features including capsid size, shape, structure, etc., as well as physicochemical and antigenic properties. As more and more viral genomes are being sequenced, the evolutionary relationship of a great many families and genera is being explored [2,3] by sequence analysis on single gene or gene families, such as polymerase, capsid and movement genes [4-10]. The virus taxonomy system is approved and updated by the International Committee on the Taxonomy of Viruses (ICTV).

However, it is full of ambiguity for phylogenetic analysis based on single gene when using conserved or similar genes since horizontal gene transfer (HGT) between viruses, along with gene duplication, gene capture from host appears to have been frequent in large DNA viruses [11-14]. Genetic mosaicism of phages has been known for a long time. Homologous morons (coding region and transcription control sequences) are found in many lineages of phages [15], and these kinds of genetic acquirements have also been considered the main sources of increasing genome size in large DNA viruses [9,16]. The high substitution rate of viruses also limits the sequence-based methods from revealing the distant evolutionary relationships [11,17]. For example, the herpes simplex virus type 1 mutates 10 times greater than mammalian genes (nearly 3.5 × 10^8 ^substitutions per site per year) [18,19]. Some attempts have been made to combine viral structure and function characteristics and genomic information of their hosts into sequence information [11,17] although quantifying such structural similarity has proved to be extremely challenging.

In the meantime there are several other attempts to infer viral phylogeny from their whole genomes [12,20-25] to avoid the problem of gene rearrangement, gene loss, gene duplication and lateral gene transfer. However, some of them infer the majority consensus tree of the many trees of individual genes or use the combined sequences of many shared genes [12,21,22]. Some of them employ gene content [12,22,23] and gene order [12,22] method, but the former has to correct for the genome size effect [26] and the latter can be hindered by a lack of synteny conservation or the variation of the evolving rate of synteny between taxa [12,26]. Above methods are partly or completely based on alignment of conserved or similar sequences which is hard to infer more distant evolutionary relationships. Gao and Stuart [20,25] apply new alignment-free methods to resolve virus relationships respectively, which appear to be sufficiently powerful to explore the phylogeny of viruses at large evolutionary distance.

Since viruses have no universal common genes just like SSU rRNA in cellular life, it is difficult to reconstruct the phylogenetic tree for distinct type of virus with most of former methods. We present here a phylogenetic analysis of large DNA viruses with the Composition Vector (CV) method [20,27,28] and discuss their relationships at a deep level. The CV method does not require extended alignments, predefined operational orthologs, or even predefined homologs (for details see material and methods). We show that the results are mostly consistent with the biologist's systematics with only a few outliers and also provide some information for those unclassified viruses and cladistic relationships of several families.

## Results and discussion

**Figure 1:**
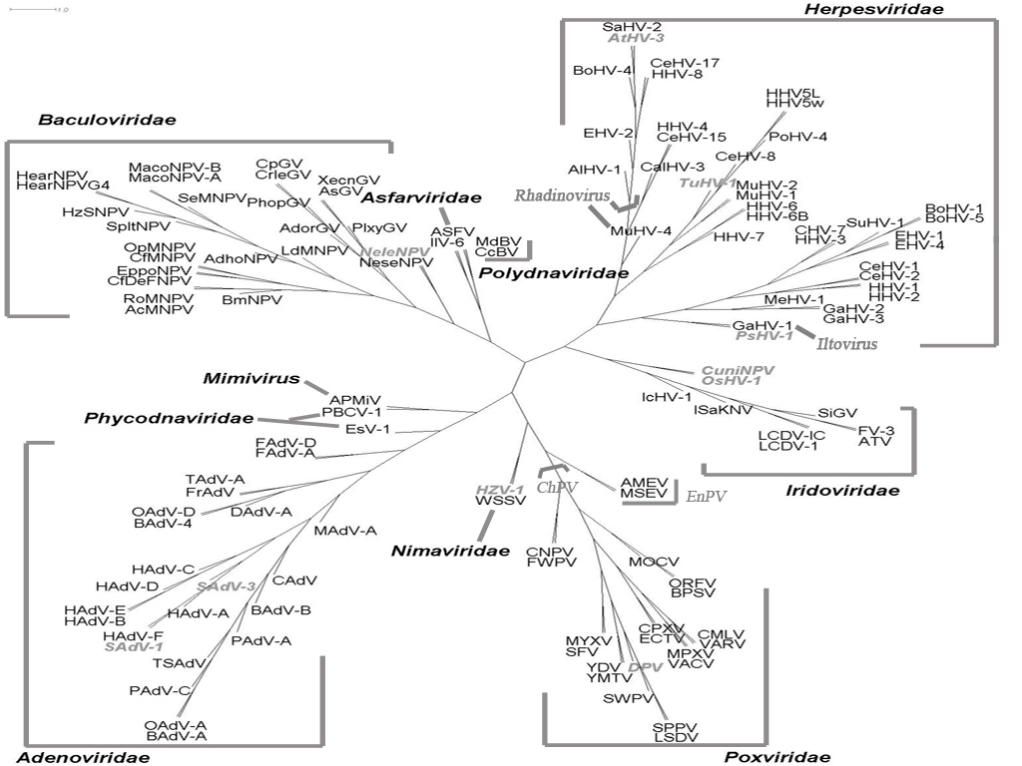
**Tree of 124 large DNA viruses**. Phylogenetic analysis of large DNA viruses based on CV method with K-string length *K *= 5. Altogether 33 genera from 10 families are presented. The nine unclassified dsDNA viruses are indicated by grey. Family names are placed close to the corresponding branches. Note that this is an unrooted tree and the branches are not to scale.

A phylogenetic tree including 124 dsDNA viruses is shown in Figure 1. Apparently, despite numerous horizontal gene transfer among large DNA viruses [13], our analysis is able to divide the 124 dsDNA viruses into 10 families with only 4 outliers, CuniNPV, IIV-6, IcHV-1 and OsHV-1 (see Additional file 1: 124 large dsDNA virus names, abbreviations, and NCBI accession numbers for viruses names, abbreviations and accession numbers).

Phylogenetic relationships of all 124 dsDNA viruses coming from 33 genera, 10 families are well consistent with the taxonomy by ICTV [1] and other phylogenetic studies [9] with few exceptions.

### Adenoviridae

**Figure 2:**
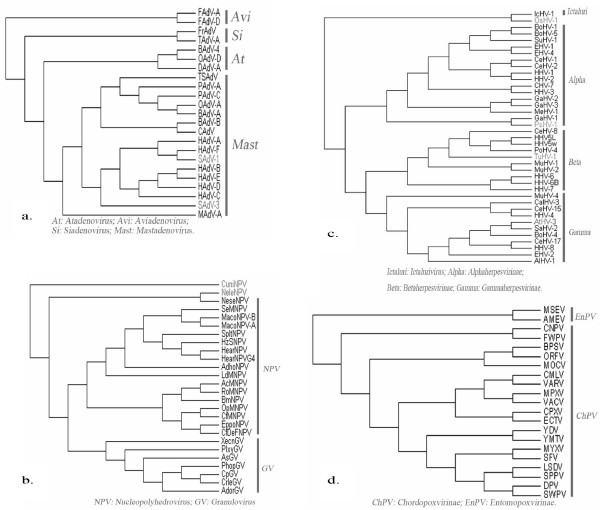
**Trees of 4 virus families**. Phylogenetic analysis of 4 virus families based on CV method with K-string length *K *= 5. a: Adenoviridae; b: Baculoviridae; c: Herpesviridae; d: Poxviridae. The unclassified dsDNA viruses are indicated by grey. Genus or subfamily names are placed close to the corresponding branches.

Fig. 1 and Fig. 2a supports the division of this family into four genera. It is notable that the two genera, *Atadenovirus *and *Siadenovirus*, which both comprise viruses from a variety of hosts locate between another two genera, *Mastadenovirus *whose hosts are mammals and *Aviadenovirus *whose hosts are birds. This variety of host origin supports the hypothesis that interspecies transmission, i.e. host switches of adenoviruses, may have occurred [29].

### Baculoviridae

According to the classification in ICTV database, one of the largest families in dsDNA viruses, *Baculoviridae*, is composed of two genera, *Granulovirus *and *Nucleopolyhedrovirus*. However, the 4-subbranch of this family shown in Fig. 1 and Fig. 2b complies with the classification of their hosts. *Dipteran*-infecting baculovirus, CuniNPV, locates the most deep [30] and stay outside the whole large clade, followed by the *Hymenoptera *baculovirus (NeleNPV and NeseNPV) and *Lepidoptera *baculovirus (the rest of them) [12,31]. There are three hypotheses on the origin of *Baculoviruses*: originated within *Lepidoptera *with subsequent horizontal transmissions to other insect orders [32]; originated with the cocladogenesis of the viruses and their hosts [33]; originated from the ancestral baculoviruses that were probably able to infect the hosts of different orders, with ancient coevolution between the hosts and pathogens then leading to the different order host specialization [12]. Our analysis apparently provides equal support to the last two hypotheses as postulated by Herniou [30], without comparing the division time of viruses and their hosts.

### Poxviridae

The division of *Poxviridae *into two subfamilies *Chordopoxvirinae *(ChPV) and *Entomopoxvirinae *(EnPV) shown in Fig. 1 and Fig. 2d, is the same as in the systematics of ICTV. Within the cluster *Orthopoxviruses *in ChPV, ECTV and CPXV are the most divergent, which is similar to McLysaght's study [34] and is also supported by another analysis based on multiple genes alignment [3]. *Capripoxvirus*, *Leporipoxvirus*, *Suipoxvirus *and *Yatapoxvirus *form another cluster, in which *Capripoxvirus *and *Suipoxvirus *are much closer to each other, and this supports the hypothesis that they might have evolved from a common ancestor [3]. In Figure 1, DPV, an unclassified *Poxvirus*, is assigned to ChPV subfamily, which agrees with Afonso's result [35].

### Herpesviridae

Within the *Herpesviridae *family, Fig. 1 and Fig. 2c also supports the observations of an early split of the *Beta- *and *Gammaherpesviruses *from the *Alphaherpesviruses *[36]. It is worth mentioning that TuHV-1, previously known only to belong to the *Beta*-subfamily, now is found to cluster with *Cytomegaloviruses *in Fig. 1, which follows Bahr's analysis [37]. According to the taxonomy system of ICTV, the *Gamma*-subfamily consists of two genera, *Lymphocryptovirus *and *Rhadinovirus*. MuHV-4, whose position was previously unresolved and various [36,38] is now assigned to *Rhadinoviruses *according to ICTV, and is the most divergent in fig. 1. The rest two ungulate herpesviruses within *Rhadinoviruses*, AIHV-1 and EHV-2, are divergent from others which is in accordance with other analyses [36,38]. However, another ungulate herpesvirus, BoHV-4, clusters closely to HHV-8 and CeHV-17, which is incompatible with the hypothesis that herpesviruses have coevolved with their hosts [39].

All *Iridoviruses *except IIV-6 fall into one cluster in fig. 1. IIV-6 and ASFV from *Asfarviridae *group together, which partly supports the theory that *Iridoviridae *and *Asfarviridae *are monophyletic [40]. It is interesting to note that ISaKNV, which was still an unclassified *Iridovirus *at the time we fixed our data sets, has been placed in a new genus *Megalocytivirus*, which supports both our analysis and Do's [41]. The same is true of PsHV-1, which is assigned to *Alphaherpesvirinae *in fig. 1 just as ICTV has done not long ago, and it should belong to *Iltovirus *for it clusters to GaHV-1. Similarly, WSSV, an unclassified marine invertebrate virus [42], has also been classified into a new virus family, *Nimaviridae*, which is again supported by our results.

Our results could also provide some clues to these hypotheses about origins and evolution of viruses of several families.

Several unclassified viruses are analyzed for obtaining some hints for their possible taxonomic statuses. As shown in fig. 1, PsHV-1 may belong to *Iltovirus*, which was supported by Thureen's result [43]; AtHV-3 to *Rhadinovirus *the same as McGeoch's analysis [44]; TuHV-1 to Cytomegalovirus just as Bahr's result [37]. SAdV-3 and SAdV-1 are close to HAdV-(A, B, C, D, E, F), and they may be two new species of *Mastadenovirus *[45,46]. NeleNPV and NeseNPV group together, and they may belong to a new genus according to Herniou's results [30]. In fig. 1 DPV locates between *Suipoxvirus *and *Yatapoxvirus *but not very closes to each of them, further supporting the idea that it appears to be assigned to a new genus *Cervidpoxvirus *[35,47]. HZV-1, originally defined as *Baculovirus *but currently as an unclassified dsDNA virus [48], clusters with WSSV whin *Nimaviridae *in our results. 

However, there are some outliers in fig. 1. IcHV-1 and OsHV-1 group closely but jump out of the branch of *Herpesviridae*, which is consistent with dissimilarities in sequence comparisons between OsHV-1 and the three vertebrate herpesvirus subfamilies [49] and up-to-date classification to two new families [50]. CuniNPV stay outside the whole large clade of *Baculoviridae *[51], and it also should belong to a new genus according to Herniou's proposal [30]. IIV-6 closes with ASFV [40] but stays outside all other *Iridoviruses*, which may support partly the theory that *Iridoviridae *and *Asfarviridae *are monophyletic [40].

## Conclusion

We present here a phylogenetic analysis of large DNA viruses with the CV method and discuss their relationships at a deep level. The results support the biologist's systematics in overall structure and in many details and provide some clues to these hypotheses about origins and evolution of viruses of several families.

It should be pointed out that although baculoviruses and their hosts are obviously subject to coevolution [30], the phylogenetic relationships of many families and the lower taxonomic levels cannot be fully explained by only the hypothesis of coevolution, e.g. the variety of host origin of *Atadenovirus *and *Siadenovirus *and the location of BoHV-4.

Some traditional methods, e.g. the measures by concatenating aligned sequences, are efficient and powerful to recover the phylogeny of virus with closely evolutionary relationship. However, definition and selection of orthologs may limit their application to distance evolutionary viruses. Furthermore, these methods, in some cases, need adjustment or fine turning.

The CV method could circumvents the ambiguity of choosing orthologs especially for viruses since substitution rate of viruses is high and only a few number of universal common genes could be found (another paper about the stable analysis of the CV method will be submitted subsequently), it may suggest a new angle to Large DNA viruses evolution. Furthermore, the CV method is robust to HGT events. It has been observed that combining many genes could reduce sampling error and converge phylogenies on correct solution with good support [12,52]. Herniou obtained 32 different tree topologies by using 63 individual genes and one tree based on the combined alignment of the 63 genes, while the latter was consistent with most individual gene trees [12]. The CV method could use the information from all coding proteins so that it may still construct stable trees even dashing with a few horizontal transferred genes. We used two sets of data in our previous analysis on bacteria: one is based on whole genomes, and the other is a set of ribosome proteins. Both the results lead to reasonable phylogenetic trees but the first one is better, this shows that these orthologs only appeared in a subset species would also help to stabilize the tree topology. In this way, the method could be a well supplement to the traditional methods. The CV method may provide a quick reference in viruses phylogeneny and a fast analysis of co-evolution of viruses and their hosts whenever their proteomes are available [26].

## Methods

All viral genomes were downloaded from NCBI before May. 24th, 2005. There are two available data sets of virus complete genomes. Those in GenBank [53] are the original data submitted by their authors. Those at the National Center for Biotechnological Information (NCBI) [54] are reference genomes curated by NCBI staff. Since the latter represents the approach of one and the same group using the same set of tools, it may provide a more consistent background for comparison. Therefore, we used all the translated amino acid sequences (the .faa files with NC_accession numbers) from NCBI. There are 1489 viral genomes, including 248 phages. Under the assumption that small DNA viruses (genome size < 10 k) probably have a different evolutionary history than large DNA viruses [9,11], and their mutation rate approaches that of RNA viruses (the order of substitutions per site per year, [55]), only large DNA viruses (total length of all coding proteins > 4 k) were used in the phylogenetic analysis, which included 124 viruses (phages have been excluded). Among the 124 dsDNA viruses there are seven viruses that are classified to certain families but their lower taxonomy states remain unknown, and two viruses are tentative species, and one virus that is only recognized as a dsDNA virus. The Additional file 1 lists the dsDNA viruses used, their abbreviations, and the NCBI accession numbers [see Additional file 1: 124 large dsDNA virus names, abbreviations, and NCBI accession numbers].

The main steps of the method are (see [28] for details): First, collect all amino acid sequences of a species. Second, calculate the frequency of appearance of overlapping oligopeptides of length *K*. A random background needs to be subtracted from these frequencies by using a Markov model of order (*K *- 2) in order to diminish the influence of random neutral mutations at the molecular level and to highlight the shaping role of selective evolution. Some strings that contribute mostly to apomorphic characters become more significant after the subtraction. The subtraction procedure is an essential step in our method. Third, putting these "normalized" frequencies in a fixed order, a composition vector of dimension 20^*K *^is obtained for each species. Fourth, the correlation *C*(*A*, *B*) between two species *A *and *B *is determined by taking projection of one normalized vector on another, i.e., taking the cosine of the angle between them. Lastly, the normalized distance between the two species is defined to be *D *= (1 - *C*)/2. Once a distance matrix has been calculated it is straightforward to construct phylogenetic trees by following the standard neighbor-joining method in the Phylip package [56].

The best choice of *K *is related to the uniqueness of sequence reconstruction from its *K*-word components and is determined basically by the length of the sequence at hand. According to so-called "sequencing by hybridization" [57], for dsDNA viral genomes with length around 4,000 a.a., the minimal *K *is estimated to be 5.

Only large genome viruses are used in our analysis to avoid the problem of small sample size when using CV method whose subtraction procedure is based on statistics. The CV method avoids the problems caused by HGT on the application of prokaryotic phylogeny by using whole genome sequences, because the extent of lateral transfer has been increasingly restricted to smaller and smaller gene pools of closer and closer related species as time goes by [58]. However, its application on classification of small DNA viruses may be affected by HGT because of relative shorter genome length, that's one of the reasons only large DNA viruses are used.

## Authors' contributions

LG carried out the molecular phylogenetic studies, participated in the design of program, and drafted the manuscript. JQ carried out the design of program and algorithm, participated in molecular phylogenetic studies, and helped to draft the manuscript.

## Supplementary Material

Additional File 1 124 large dsDNA virus names, abbreviations, and NCBI accession numbers. Click here for file

## References

[B1] van Regenmortel MHV, Fauquet CM, Bishop DHL, Carstens EB, Estes MK, Lemon SM, Maniloff J, Mayo MA, McGeoch DJ, Pringle CR, Wickner RB (2000). Virus Taxonomy: Seventh Report of the International Committee on Taxonomy of Viruses.

[B2] Davison AJ, Benko M, Harrach B (2003). Genetic content and evolution of adenoviruses. J Gen Virol.

[B3] Gubser C, Hue S, Kellam P, Smith GL (2004). Poxvirus genomes: a phylogenetic analysis. J Gen Virol.

[B4] Bulach DM, Kumar CA, Zaia A, Liang B, Tribe DE (1999). Group II nucleopolyhedrovirus subgroups revealed by phylogenetic analysis of polyhedrin and DNA polymerase gene sequences. J Invertebr Pathol.

[B5] Chen X, Ijkel WFJ, Dominy C, Zanotto P, Hashimoto Y, Faktor O, Hayakawa T, Wang CH, Prekumar A, Mathavan S, Krell PJ, Hu Z, Vlak JM (1999). Identification, sequence analysis and phylogeny of the lef-2 gene of Helicoverpa armigera single-nucleocapsid baculovirus. Virus Res.

[B6] Koonin EV (1991). The phylogeny of RNA-dependent RNA polymerases of positive-strand RNA viruses. Journal of General Virology.

[B7] Melcher U (2000). The '30K' superfamily of viral movement proteins. Journal of General Virology.

[B8] Tetart F, Desplats C, Kutateladze M, Monod C, Ackermann HW, Krisch HM (2001). Phylogeny of the major head and tail genes of the wide-ranging T4-Type bacteriophage. J Bacteriol.

[B9] Tidona CA, Darai G (2000). Iridovirus homologues of cellular genes: implications for the molecular evolution of large DNA viruses. Virus Genes.

[B10] Tidona CA, Schnitzler P, Kehm R, Darai G (1998). Is the major capsid protein of Iridoviruses a suitable target for the study of viral evolution?. Virus genes.

[B11] Shackelton LA, Holmes EC (2004). The evolution of large DNA viruses: combining genomic information of viruses and their hosts. Trends in Microbiology.

[B12] Herniou EA, Luque T, Chen X, Vlak JM, Winstanley D, Cory JS, O'Reilly DR (2001). Use of whole genome sequence data to infer baculovirus phylogeny. Journal of Virology.

[B13] Filee J, Forterre P, Laurent J (2003). The role played by viruses in the evolution of their hosts: a view based on informational protein phylogenies. Res Microbiol.

[B14] Hughes AL (2002). Origin and evolution of viral interleukin-10 and other DNA virus genes with vertebrate homologues. J Mol Evol.

[B15] Hendrix RW, Lawrence JG, Hatfull GF, Casjens S (2000). The origins and ongoing evolution of viruses. Trends in Microbiology.

[B16] Bugert JJ, Darai G (2000). Poxvirus homologues of cellular genes. Virus Genes.

[B17] Bamford DH, Burnett RM, Stuart D (2002). Evolution of viral structure. Theoretical Population Biology.

[B18] Li W (1997). Molecular Evolution.

[B19] Sakaoka H, Kurita K, Iida Y, Takada S, Umene K, Kim YT, Ren CS, Nahmias AJ (1994). Quantitative analysis of genomic polymorphism of herpes simplex virus type 1 strains from six countries: studies of molecular evolution and molecular epidemiology of the virus. J Gen Virol.

[B20] Gao L, Qi J, Wei H, Sun Y, Hao B (2003). Molecular phylogeny of coronaviruses including human SARS-CoV. Chinese Science Bulletin.

[B21] Harrison RL, Bonning BC (2003). Comparative analysis of the genomes of Rachiplusiaou and Autographa californica multiple nucleopolyhedroviruses. J Gen Virol.

[B22] Hyink O, Dellow RA, Olsen MJ, Caradoc-Davies KMB, Drake K, Herniou EA, Cory JS, O'Reilly DR, Ward VK (2002). Whole genome analysis of the Epiphyas postvittana nucleopolyhedrovirus. J Gen Virol.

[B23] Montague MG, Hutchison CA (2000). Gene content phylogeny of herpesviruses. Proc Natl Acad Sci USA.

[B24] Rohwer F, Edwards R (2002). The phage proteomic tree: a genome-based taxonomy for phage. Journal of Bacteriology.

[B25] Stuart G, Moffett K, Bozarth RF (2004). A whole genome perspective on the phylogeny of the plant virus family Tombusviridae. Archives of Virology.

[B26] Snel B, Huynen MA, Dutilh BE (2005). Genome trees and the nature of genome evolution. Annu Rev Microbiol.

[B27] Chu K, Qi J, Yu Z, Anh V (2004). Origin and Phylogeny of Chloroplasts Revealed by a Simple Correlation Analysis of Complete Genomes. Mol Biol Evol.

[B28] Qi J, Wang B, Hao B (2004). Whole Proteome Prokaryote Phylogeny without Sequence Alignment: A K-String Composition Approach. Journal of Molecular Evolution.

[B29] Farkas SL, Benkö M, Élö P, Ursu K, Dán Á, Ahne W, Harrach B (2002). Genomic and phylogenetic analyses of an adenovirus isolated from a corn snake (Elaphe guttata) imply common origin with the members of the proposed new genus Atadenovirus. J Gen Virol.

[B30] Herniou EA, Olszewski JA, O'Reilly DR, Cory JS (2004). Ancient coevolution of baculoviruses and their insect hosts. Journal of Virology.

[B31] Zanotto PM, Kessing BD, Maruniak JE (1993). Phylogenetic interrelationships among baculoviruses: evolutionary rates and host associations. Journal of Invertebrate Phathology.

[B32] Rohrmann GF, Granados R, Federici B (1986). Evolution of occluded baculoviruses. The biology of baculoviruses.

[B33] Federici BA, Miller LK (1997). Baculovirus pathogenesis. The baculoviruses.

[B34] McLysaght A, Baldi PF, Gaut BS (2003). Extensive gene gain associated with adaptive evolution of poxviruses. Proc Natl Acad Sci USA.

[B35] Afonso CL, Delhon G, Tulman ER, Lu Z, Zsak A, Becerra VM, Zsak L, Kutish GF, Roch DL (2005). Genome of Deerpox Virus. J Virol.

[B36] Albà MM, Das R, Orengo CA, Kellam P (2001). Genomewide function conservation and phylogeny in the Herpesviridae. Genome Res.

[B37] Bahr U, Darai G (2001). Analysis and characterization of the complete genome of tupaia (tree shrew) herpesvirus. J Virol.

[B38] McGeoch DJ, Davison AJ (1999). The descent of human herpesvirus 8. Seminars in Cancer Biology.

[B39] McGeoch DJ, Cook S (1994). Molecular phylogeny of the Alphaherpesvirinae subfamily and a proposed evolutionary timescale. J Mol Biol.

[B40] Iyer LM, Aravind L, Koonin EV (2001). Common origin of four diverse families of large eukaryotic DNA viruses. J Virol.

[B41] Do JW, Moon CH, Kim HJ, Ko MS, Kim SB, Son JH, Kim JS, An EJ, Kim MK, Lee SK, Han MS, Cha SJ, Park MS, Park MA, Kim YC, Kim JW, Park JW (2004). Complete genomic DNA sequence of rock bream iridovirus. Virology.

[B42] Yang F, He J, Lin X, Pan D, Zhang X, Xu X (2001). Complete genome sequence of the shrimp white spot bacilliform virus. J Virol.

[B43] Thureen DR, Keeler CL (2006). Psittacid Herpesvirus 1 and Infectious Laryngotracheitis Virus: Comparative Genome Sequence Analysis of Two Avian Alphaherpesviruses. J Virol.

[B44] McGeoch DJ, Gatherer D, Dolan A (2005). On phylogenetic relationships among major lineages of the Gammaherpesvirinae. J Gen Virol.

[B45] Kovács GM, Davison AJ, Zakhartchouk AN, Harrach B (2004). Analysis of the first complete genome sequence of an Old World monkey adenovirus reveals a lineage distinct from the six human adenovirus species. J Gen Virol.

[B46] Kovács GM, Harrach B, Zakhartchouk AN, Davison AJ (2005). Complete genome sequence of simian adenovirus 1: an Old World monkey adenovirus with two fiber genes. J Gen Virol.

[B47] Lefkowitz EJ, Wang C, Upton C (2006). Poxviruses: past, present and future. Virus Research.

[B48] Cheng CH, Liu SM, Chow TY, Hsiao YY, Wang DP, Huang JJ, Chen HH (2002). Analysis of the complete genome sequence of the Hz-1 virus suggests that it is related to members of the Baculoviridae. J Virol.

[B49] Davison AJ, Trus BL, Cheng N, Steven AC, Watson MS, Cunningham C, Le Deuff RM, Renault T (2005). A novel class of herpesvirus with bivalve hosts. J Gen Virol.

[B50] McGeoch DJ, Rixon FJ, Davison AJ (2006). Topics in herpesvirus genomics and evolution. Virus Research.

[B51] Moser BA, Becnel JJ, White SE, Afonso C, Kutish G, Shanker S, Almira E (2001). Morphological and molecular evidence that Culex nigripalpus baculovirus is an unusual member of the family Baculoviridae. J Gen Virol.

[B52] Mitchell A, Mitter C, Regier JC (2000). More taxa or more characters revisited: combining data from nuclear protein-encoding genes for phylogenetic analyses of Noctuoidea (Insecta: Lepidoptera). Syst Biol.

[B53] Benson DA, Karsch-Mizrachi I, Lipman DJ, Ostell J, Wheeler DL (2006). GenBank. Nucleic Acids Res.

[B54] Wheeler DL, Barrett T, Benson DA, Bryant SH, Canese K, Chetvernin V, Church DM, DiCuccio M, Edgar R, Federhen S, Geer LY, Helmberg W, Kapustin Y, Kenton DL, Khovayko O, Lipman DJ, Madden TL, Maglott DR, Ostell J, Pruitt KD, Schuler GD, Schriml LM, Sequeira E, Sherry ST, Sirotkin K, Souvorov A, Starchenko G, Suzek TO, Tatusov R, Tatusova TA, Wagner L, Yaschenko E (2006). Database resources of the National Center for Biotechnology Information. Nucleic Acids Res.

[B55] Truyen U, Gruenberg A, Chang S, Obermaier B, Veijalainen P, Parrish C (1995). Evolution of the feline-subgroup parvoviruses and the control of canine host-range in vivo. J Virol.

[B56] Felsenstein J PHYLIP (phylogeny inference package) version 3.5c. http://evolution.genetics.washington.edu/phylip.html.

[B57] Pevzner PA (2000). Computational Molecular Biology: An Algorithmic Approach.

[B58] Woese CR (1998). The universal ancester. Proc Natl Acad Sci USA.

