# Supplementary material for: Whole genome molecular phylogeny of large dsDNA viruses using composition vector method
Source: BMC Evol Biol. 2007 Mar 15;7:41. doi: 10.1186/1471-2148-7-41 (PMC1839080; doi:10.1186/1471-2148-7-41)
Supplement: Additional File 1 — 124 large dsDNA virus names, abbreviations, and NCBI accession numbers. [file 1471-2148-7-41-S1.doc]

Additional file 1. 124 large dsDNA virus names, abbreviations, and NCBI accession numbers.

| Species | Abbr. | Accession | Family | Genus |
| --- | --- | --- | --- | --- |
| Bovine adenovirus D | BAdV-4 | NC_002685.2 | *Adenoviridae* | Atadenovirus |
| Ovine adenovirus D | OAdV-D | NC_004037.1 | *Adenoviridae* | Atadenovirus |
| Duck adenovirus A | DAdV-A | NC_001813.1 | *Adenoviridae* | Atadenovirus |
| Fowl adenovirus A | FAdV-A | NC_001720.1 | *Adenoviridae* | Aviadenovirus |
| Fowl adenovirus D | FAdV-D | NC_000899.1 | *Adenoviridae* | Aviadenovirus |
| Bovine adenovirus B | BAdV-B | NC_001876.1 | *Adenoviridae* | Mastadenovirus |
| Canine adenovirus | CAdV | NC_001734.1 | *Adenoviridae* | Mastadenovirus |
| Human adenovirus A | HAdV-A | NC_001460.1 | *Adenoviridae* | Mastadenovirus |
| Human adenovirus B | HAdV-B | NC_004001.2 | *Adenoviridae* | Mastadenovirus |
| Human adenovirus C | HAdV-C | NC_001405.1 | *Adenoviridae* | Mastadenovirus |
| Human adenovirus D | HAdV-D | NC_002067.1 | *Adenoviridae* | Mastadenovirus |
| Human adenovirus E | HAdV-E | NC_003266.2 | *Adenoviridae* | Mastadenovirus |
| Murine adenovirus A | MAdV-A | NC_000942.1 | *Adenoviridae* | Mastadenovirus |
| Ovine adenovirus A | OAdV-A | NC_002513.1 | *Adenoviridae* | Mastadenovirus |
| Porcine adenovirus C | PAdV-C | NC_002702.1 | *Adenoviridae* | Mastadenovirus |
| Simian adenovirus A | SAdV-3 | NC_006144.1 | *Adenoviridae* | Mastadenovirus |
| Bovine adenovirus A | BAdV-A | NC_006324.1 | *Adenoviridae* | Mastadenovirus |
| Human adenovirus F | HAdV-F | NC_001454.1 | *Adenoviridae* | Mastadenovirus |
| Porcine adenovirus A | PAdV-A | NC_005869.1 | *Adenoviridae* | Mastadenovirus |
| Tree shrew adenovirus | TSAdV | NC_004453.1 | *Adenoviridae* | Mastadenovirus |
| Simian adenovirus | SAdV-1 | NC_006879.1 | *Adenoviridae* | Mastadenovirus |
| Frog adenovirus | FrAdV | NC_002501.1 | *Adenoviridae* | Siadenovirus |
| Turkey adenovirus A | TAdV-A | NC_001958.1 | *Adenoviridae* | Siadenovirus |
| African swine fever virus | ASFV | NC_001659.1 | *Asfarviridae* | Asfivirus |
| Adoxophyes orana granulovirus | AdorGV | NC_005038.1 | *Baculoviridae* | Granulovirus |
| Agrotis segetum granulovirus | AsGV | NC_005839.2 | *Baculoviridae* | Granulovirus |
| Cryptophlebia leucotreta granulovirus | CrleGV | NC_005068.1 | *Baculoviridae* | Granulovirus |
| Cydia pomonella granulovirus | CpGV | NC_002816.1 | *Baculoviridae* | Granulovirus |
| Phthorimaea operculella granulovirus | PhopGV | NC_004062.1 | *Baculoviridae* | Granulovirus |
| Plutella xylostella granulovirus | PlxyGV | NC_002593.1 | *Baculoviridae* | Granulovirus |
| Xestia c-nigrum granulovirus | XecnGV | NC_002331.1 | *Baculoviridae* | Granulovirus |
| Adoxophyes honmai nucleopolyhedrovirus | AdhoNPV | NC_004690.1 | *Baculoviridae* | Nucleopolyhedrovirus |
| Autographa californica nucleopolyhedrovirus | AcMNPV | NC_001623.1 | *Baculoviridae* | Nucleopolyhedrovirus |
| Bombyx mori nucleopolyhedrovirus | BmNPV | NC_001962.1 | *Baculoviridae* | Nucleopolyhedrovirus |
| Choristoneura fumiferana defective nucleopolyhedrovirus | CfDeFNPV | NC_005137.2 | *Baculoviridae* | Nucleopolyhedrovirus |
| Choristoneura fumiferana MNPV | CfMNPV | NC_004778.3 | *Baculoviridae* | Nucleopolyhedrovirus |
| Epiphyas postvittana nucleopolyhedrovirus | EppoNPV | NC_003083.1 | *Baculoviridae* | Nucleopolyhedrovirus |
| Helicoverpa armigera nuclear polyhedrosis virus | HearNPV | NC_003094.2 | *Baculoviridae* | Nucleopolyhedrovirus |
| Helicoverpa armigera nucleopolyhedrovirus G4 | HearNPVG4 | NC_002654.1 | *Baculoviridae* | Nucleopolyhedrovirus |
| Helicoverpa zea single nucleocapsid nucleopolyhedrovirus | HzSNPV | NC_003349.1 | *Baculoviridae* | Nucleopolyhedrovirus |
| Lymantria dispar nucleopolyhedrovirus | LdMNPV | NC_001973.1 | *Baculoviridae* | Nucleopolyhedrovirus |
| Mamestra configurata nucleopolyhedrovirus A | MacoNPV-A | NC_003529.1 | *Baculoviridae* | Nucleopolyhedrovirus |
| Mamestra configurata nucleopolyhedrovirus B | MacoNPV-B | NC_004117.1 | *Baculoviridae* | Nucleopolyhedrovirus |
| Neodiprion sertifer nucleopolyhedrovirus | NeseNPV | NC_005905.1 | *Baculoviridae* | Nucleopolyhedrovirus |
| Orgyia pseudotsugata multicapsid nucleopolyhedrovirus | OpMNPV | NC_001875.2 | *Baculoviridae* | Nucleopolyhedrovirus |
| Rachiplusia ou multiple nucleopolyhedrovirus | RoMNPV | NC_004323.1 | *Baculoviridae* | Nucleopolyhedrovirus |
| Spodoptera exigua nucleopolyhedrovirus | SeMNPV | NC_002169.1 | *Baculoviridae* | Nucleopolyhedrovirus |
| Spodoptera litura nucleopolyhedrovirus | SpltNPV | NC_003102.1 | *Baculoviridae* | Nucleopolyhedrovirus |
| Culex nigripalpus baculovirus | CuniNPV | NC_003084.1 | *Baculoviridae* | unclassified Baculoviridae |
| Neodiprion lecontei nucleopolyhedrovirus | NeleNPV | NC_005906.1 | *Baculoviridae* | unclassified Baculoviridae |
| Gallid herpesvirus 1 | GaHV-1 | NC_006623.1 | *Herpesviridae* | Iltovirus |
| Gallid herpesvirus 2 | GaHV-2 | NC_002229.2 | *Herpesviridae* | Mardivirus |
| Gallid herpesvirus 3 | GaHV-3 | NC_002577.1 | *Herpesviridae* | Mardivirus |
| Meleagrid herpesvirus 1 | MeHV-1 | NC_002641.1 | *Herpesviridae* | Mardivirus |
| Cercopithecine herpesvirus 1 | CeHV-1 | NC_004812.1 | *Herpesviridae* | Simplexvirus |
| Human herpesvirus 1 | HHV-1 | NC_001806.1 | *Herpesviridae* | Simplexvirus |
| Human herpesvirus 2 | HHV-2 | NC_001798.1 | *Herpesviridae* | Simplexvirus |
| Cercopithecine herpesvirus 2 | CeHV-2 | NC_006560.1 | *Herpesviridae* | Simplexvirus |
| Bovine herpesvirus 1 | BoHV-1 | NC_001847.1 | *Herpesviridae* | Varicellovirus |
| Bovine herpesvirus 5 | BoHV-5 | NC_005261.1 | *Herpesviridae* | Varicellovirus |
| Cercopithecine herpesvirus 9 | CHV-7 | NC_002686.1 | *Herpesviridae* | Varicellovirus |
| Equid herpesvirus 1 | EHV-1 | NC_001491.2 | *Herpesviridae* | Varicellovirus |
| Equid herpesvirus 4 | EHV-4 | NC_001844.1 | *Herpesviridae* | Varicellovirus |
| Suid herpesvirus 1 | SuHV-1 | NC_006151.1 | *Herpesviridae* | Varicellovirus |
| Human herpesvirus 3 (strain Dumas) | HHV-3 | NC_001348.1 | *Herpesviridae* | Varicellovirus |
| Human herpesvirus 5 strain AD169 | HHV5L | NC_001347.2 | *Herpesviridae* | Cytomegalovirus |
| Human herpesvirus 5 strain Merlin | HHV5w | NC_006273.1 | *Herpesviridae* | Cytomegalovirus |
| Pongine herpesvirus 4 | PoHV-4 | NC_003521.1 | *Herpesviridae* | Cytomegalovirus |
| Cercopithecine herpesvirus 8 | CeHV-8 | NC_006150.1 | *Herpesviridae* | Cytomegalovirus |
| Murid herpesvirus 1 | MuHV-1 | NC_004065.1 | *Herpesviridae* | Muromegalovirus |
| Murid herpesvirus 2 | MuHV-2 | NC_002512.2 | *Herpesviridae* | Muromegalovirus |
| Human herpesvirus 6 | HHV-6 | NC_001664.1 | *Herpesviridae* | Roseolovirus |
| Human herpesvirus 6B | HHV-6B | NC_000898.1 | *Herpesviridae* | Roseolovirus |
| Human herpesvirus 7 | HHV-7 | NC_001716.2 | *Herpesviridae* | Roseolovirus |
| Tupaiid herpesvirus 1 | TuHV-1 | NC_002794.1 | *Herpesviridae* | unclassified Betaherpesvirinae |
| Callitrichine herpesvirus 3 | CalHV-3 | NC_004367.1 | *Herpesviridae* | Lymphocryptovirus |
| Human herpesvirus 4 | HHV-4 | NC_001345.1 | *Herpesviridae* | Lymphocryptovirus |
| Cercopithecine herpesvirus 15 | CeHV-15 | NC_006146.1 | *Herpesviridae* | Lymphocryptovirus |
| Cercopithecine herpesvirus 17 | CeHV-17 | NC_003401.1 | *Herpesviridae* | Rhadinovirus |
| Alcelaphine herpesvirus 1 | AIHV-1 | NC_002531.1 | *Herpesviridae* | Rhadinovirus |
| Bovine herpesvirus 4 | BoHV-4 | NC_002665.1 | *Herpesviridae* | Rhadinovirus |
| Equid herpesvirus 2 | EHV-2 | NC_001650.1 | *Herpesviridae* | Rhadinovirus |
| Human herpesvirus 8 | HHV-8 | NC_003409.1 | *Herpesviridae* | Rhadinovirus |
| Murid herpesvirus 4 | MuHV-4 | NC_001826.1 | *Herpesviridae* | Rhadinovirus |
| Saimiriine herpesvirus 2 | SaHV-2 | NC_001350.1 | *Herpesviridae* | Rhadinovirus |
| Ictalurid herpesvirus 1 | IcHV-1 | NC_001493.1 | *Herpesviridae* | Ictalurivirus |
| Ostreid herpesvirus 1 | OsHV-1 | NC_005881.1 | *Herpesviridae* | Unassigned Herpesviridae |
| Psittacid herpesvirus 1 | PsHV-1 | NC_005264.1 | *Herpesviridae* | Unassigned Herpesviridae |
| Ateline herpesvirus 3 | AtHV-3 | NC_001987.1 | *Herpesviridae* | unclassified Herpesviridae |
| Invertebrate iridescent virus 6 | IIV-6 | NC_003038.1 | *Iridoviridae* | Iridovirus |
| Lymphocystis disease virus - isolate China | LCDV-IC | NC_005902.1 | *Iridoviridae* | Lymphocystivirus |
| Lymphocystis disease virus 1 | LCDV-1 | NC_001824.1 | *Iridoviridae* | Lymphocystivirus |
| Infectious spleen and kidney necrosis virus | ISaKNV | NC_003494.1 | *Iridoviridae* | Megalocytivirus |
| Frog virus 3 | FV-3 | NC_005946.1 | *Iridoviridae* | Ranavirus |
| Regina ranavirus | ATV | NC_005832.1 | *Iridoviridae* | Ranavirus |
| Singapore grouper iridovirus | SiGV | NC_006549.1 | *Iridoviridae* | Ranavirus |
| Shrimp white spot syndrome virus | WSSV | NC_003225.1 | *Nimaviridae* | Whispovirus |
| Paramecium bursaria Chlorella virus 1 | PBCV-1 | NC_000852.3 | *Phycodnaviridae* | Chlorovirus |
| Ectocarpus siliculosus virus | EsV-1 | NC_002687.1 | *Phycodnaviridae* | Phaeovirus |
| Cotesia congregata virus | CcBV | NC_006633-62.1 | *Polydnaviridae* | Bracovirus |
| Microplitis demolitor bracovirus | MdBV | NC_007028-41.1 | *Polydnaviridae* | Bracovirus |
| Canarypox virus | CNPV | NC_005309.1 | *Poxviridae* | Avipoxvirus |
| Fowlpox virus | FWPV | NC_002188.1 | *Poxviridae* | Avipoxvirus |
| Lumpy skin disease virus | LSDV | NC_003027.1 | *Poxviridae* | Capripoxvirus |
| Sheeppox virus | SPPV | NC_004002.1 | *Poxviridae* | Capripoxvirus |
| Myxoma virus | MYXV | NC_001132.2 | *Poxviridae* | Leporipoxvirus |
| Rabbit fibroma virus | SFV | NC_001266.1 | *Poxviridae* | Leporipoxvirus |
| Molluscum contagiosum virus | MOCV | NC_001731.1 | *Poxviridae* | Molluscipoxvirus |
| Camelpox virus | CMLV | NC_003391.1 | *Poxviridae* | Orthopoxvirus |
| Cowpox virus | CPXV | NC_003663.2 | *Poxviridae* | Orthopoxvirus |
| Ectromelia virus | ECTV | NC_004105.1 | *Poxviridae* | Orthopoxvirus |
| Monkeypox virus | MPXV | NC_003310.1 | *Poxviridae* | Orthopoxvirus |
| Vaccinia virus | VACV | NC_001559.1 | *Poxviridae* | Orthopoxvirus |
| Variola virus | VARV | NC_001611.1 | *Poxviridae* | Orthopoxvirus |
| Bovine papular stomatitis virus | BPSV | NC_005337.1 | *Poxviridae* | Parapoxvirus |
| Orf virus | ORFV | NC_005336.1 | *Poxviridae* | Parapoxvirus |
| Swinepox virus | SWPV | NC_003389.1 | *Poxviridae* | Suipoxvirus |
| Yaba monkey tumor virus | YMTV | NC_005179.1 | *Poxviridae* | Yatapoxvirus |
| Yaba-like disease virus | YDV | NC_002642.1 | *Poxviridae* | Yatapoxvirus |
| Amsacta moorei entomopoxvirus | AMEV | NC_002520.1 | *Poxviridae* | Betaentomopoxvirus |
| Melanoplus sanguinipes entomopoxvirus | MSEV | NC_001993.1 | *Poxviridae* | Betaentomopoxvirus |
| Mule deer poxvirus | DPV | NC_006966.1 | *Poxviridae* | unclassified Poxviridae |
| Acanthamoeba polyphaga mimivirus | APMiV | NC_006450.1 |  | Mimivirus |
| Heliothis zea virus 1 | HZV-1 | NC_004156.1 |  | unclassified dsDNA viruses |
